# Biomarker dynamics affecting neoadjuvant therapy response and outcome of HER2-positive breast cancer subtype

**DOI:** 10.1038/s41598-023-40071-2

**Published:** 2023-08-08

**Authors:** Sandra Orrù, Emanuele Pascariello, Barbara Pes, Vincenzo Rallo, Raffaele Barbara, Marta Muntoni, Francesca Notari, Gianfranco Fancello, Cristina Mocci, Maria Rosaria Muroni, Paolo Cossu-Rocca, Andrea Angius, Maria Rosaria De Miglio

**Affiliations:** 1 Department of Pathology, “A. Businco” Oncologic Hospital, ARNA S Brotzu, Via Edward Jenner 1, 09121 Cagliari, Italy; 2 Dipartimento di Matematica e Informatica, University of Cagliari, Palazzo delle Scienze, Via Ospedale 72, 09124 Cagliari, Italy; 3 Istituto di Ricerca Genetica e Biomedica (IRGB), Consiglio Nazionale delle Ricerche, CNR, Cittadella Universitaria di Cagliari, 09042 Monserrato, Cagliari Italy; 4 Department of Radiotherapy, “A. Businco” Oncologic Hospital, ARNAS Brotzu, Via Edward Jenner 1, 09121 Cagliari, Italy; 5 Breast Surgery Department, “A. Businco” Oncologic Hospital, ARNAS Brotzu, Via Edward Jenner 1, 09121 Cagliari, Italy; 6 Department of Medicine, Surgery and Pharmacy, University of Sassari, Via P. Manzella 4, 07100 Sassari, Italy; 7 Department of Diagnostic Services, “Giovanni Paolo II” Hospital, ASSL Olbia-ATS Sardegna, Via Bazzoni-Sircana, 07026 Olbia, Italy

**Keywords:** Cancer, Biomarkers

## Abstract

HER2+ breast cancer (BC) is an aggressive subtype genetically and biologically heterogeneous. We evaluate the predictive and prognostic role of HER2 protein/gene expression levels combined with clinico-pathologic features in 154 HER2+ BCs patients who received trastuzumab-based neoadjuvant chemotherapy (NACT). The tumoral pathological complete response (pCR) rate was 40.9%. High tumoral pCR show a scarce mortality rate vs subjects with a lower response. 93.7% of ypT0 were HER2 IHC3+ BC, 6.3% were HER2 IHC 2+/SISH+ and 86.7% of ypN0 were HER2 IHC3+, the remaining were HER2 IHC2+/SISH+. Better pCR rate correlate with a high percentage of infiltrating immune cells and right-sided tumors, that reduce distant metastasis and improve survival, but no incidence difference. HER2 IHC score and laterality emerge as strong predictors of tumoral pCR after NACT from machine learning analysis. HER2 IHC3+ and G3 are poor prognostic factors for HER2+ BC patients, and could be considered in the application of neoadjuvant therapy. Increasing TILs concentrations, lower lymph node ratio and lower residual tumor cellularity are associated with a better outcome. The immune microenvironment and scarce lymph node involvement have crucial role in clinical outcomes. The combination of all predictors might offer new options for NACT effectiveness prediction and stratification of HER2+ BC during clinical decision-making.

## Introduction

HER2 is a membrane glycoprotein with tyrosine kinase activity and a member of the epidermal growth factor receptor family. HER2 protein overexpression is observed in ~ 15–20% of all invasive breast cancer (BC), and it is associated to high invasiveness and worse prognosis if not properly treated^1^. HER2 signaling triggers PI3K/AKT and RAS/RAF/MAPK pathways, boosting cell proliferation, growth, invasion, and angiogenesis in tumors^2^. HER2-targeted therapies (i.e. trastuzumab) have established a considerable upgrade in outcomes of patients affected by HER2+ BC subtype and modified the tumor natural biology^3–5^. Currently, a combination of sequential chemotherapy and anti-HER2 drug is the landmark for patients with HER2+ BC subtype, both in the neoadjuvant, adjuvant and metastatic setting^6,7^. Chemotherapy, combined with dual anti-HER2 therapies, have been used as neoadjuvant treatment in HER2+ BC patients to improve the pathological complete response (pCR) rate^7^ which is predictive for more favorable long-term outcome^8,9^.

Unfortunately, not all HER2+ BC patients have clinical improvement from anti-HER2 therapies. Several clinical trials proved that about 25–30% of HER2+ BC patients, treated with trastuzumab, after adjuvant chemotherapy experienced recurrence within 10 years of diagnosis^5,10^ and locoregional or distant recurrence has been identified after dual HER2-targeted neoadjuvant treatment (about 7.1% of cases)^11^. Identifying accurate and reliable biomarkers to predict response to HER2-targeted therapy in this subtype of BC has become a priority. The treatment with anti-HER2 drugs in BC patients is primarily based on HER2 protein overexpression established by immunohistochemistry (IHC) method, while “dual-probe in situ” hybridization (SISH) is used in case of HER2 with equivocal immunohistochemical score (IHC 2+).

The aim of the present study is the retrospective evaluation of the predictive and prognostic role of HER2 expression levels in HER2+ BC patients receiving neoadjuvant treatment together with other clinico-pathologic variables as tumor infiltrating lymphocytes (TILs), histologic grade, residual tumor cellularity (RTC). Given the fast-growing number of data mining applications reported in healthcare and clinical decision-making capable of automatically extracting meaningful patterns from the available data, we decided to integrate the results obtained through classical methods with a machine learning approach.

## Results

### Correlation between clinico-pathological parameters, HER2 score and NACT-response in HER2+ BC subtype

A total of 154 HER2+ BC patients who underwent neoadjuvant therapy and surgery were selected. The median (range) age at diagnosis was 54 (26–81) years, with 80 patients (51.9%) equal to or older than 54 years. One hundred and thirty (84.41%) tumors were ductal, 9 (5.84%) lobular, and 9 (5.84%) apocrine; less common were other histologic types, such as mucinous (2.6%) and micropapillary/papillary (1.3%). Seventy-nine (51.3%) tumors were right-sided, while 75 (48.7%) left-sided. Table 1 shows the clinico-pathological parameters of the study cohort by the HER2 score, including 120 (77.9%) BC patients who had HER2 IHC 3+, and 34 (22.1%) had HER2 IHC 2+/SISH+. The comparison between the HER2 categories showed the following features: ypT0, ypT1a and ypT1b were found in 93.7%, 69.2% and 76.9% of HER2 IHC 3+ BC vs. 6.3%, 30.8% and 23.1% of HER2 IHC 2+/SISH+ BC, respectively (P = 0.003). ypN0 and ypN1 were found in 86.7% and 74.3% of HER2 IHC 3+ BC vs 13.3% and 25.7% of HER2 IHC 2+/SISH+ BC, respectively (P < 0.001). HER2 IHC 3+ BC showed a predominantly low LNR ≤ 0.20 (P = 0.015), low post-NACT ki-67 (≤ 20%, P = 0.039), and post-NACT prognostic stage was mainly 0, IA–B or IIA (P = 0.009). HER2 IHC 3+ BC showed a RTC ≤ 5% in the 90.1% vs 9.9% in the HER2 IHC 2+/SISH+ BC (P < 0.001). HER2 IHC 3+ BC was more frequently associated with negative pre-NACT ER and PR expression (< 1% for both; P = 0.002 and P < 0.001, respectively). Finally, HER2 IHC 3+ BC showed pCR in the 93.7% of tumors and in the 86.7% of lymph node after neoadjuvant therapy vs 6.3% and 13.3% of HER2 IHC 2+/SISH+ BC, respectively (both P < 0.001).

**Table 1:** Clinico-pathological data of 154 patients with HER2-positive breast cancer based on of HER2 IHC 3+ and HER2 IHC 2+/SISH+ categories.

		HER2 IHC 3+ (n = 120) *n* (%)	HER2 IHC 2+/ISH+ (n = 34) *n* (%)	P value
Age (year)	< 54	61 (82.4)	13 (17.6)	0.194
	≥ 54	59 (73.8)	21 (26.2)	
Site	Left	57 (76.0)	18 (24.0)	0.575
	Right	63 (79.7)	16 (20.3)	
Histologic type pre-NACT	NST	100 (76.9)	30 (23.1)	0.470
	ILC	7 (77.8)	2 (22.2)	
	APOCRINE	9 (100.0)	0 (0.0)	
	MICROPAPILLARY	1 (50.0)	1 (5.0)	
	MUCINOUS	3 (75.0)	1 (25.0)	
Histologic grade pre-NACT	G2	18 (69.2)	8 (30.8)	0.241
	G3	102 (79.7)	26 (20.3)	
Tumor size (ypT)	ypT0	59 (93.7)	4 (6.3)	**0.003**
	ypT1a	9 (69.2)	4 (30.8)	
	ypT1b	10 (76.9)	3 (23.1)	
	ypT1c	12 (60.0)	8 (40.0)	
	ypT2	19 (59.4)	13 (40.6)	
	ypT3	5 (83.3)	1 (16.7)	
	ypT4	6 (85.7)	1 (14.3)	
Lymph node status (ypN)	ypN0	85 (86.7)	13 (13.3)	**0.001**
	ypN1	26 (74.3)	9 (25.7)	
	ypN2	6 (42.9)	8 (57.1)	
	ypN3	3 (42.9)	4 (57.1)	
Lymph node ratio post-NACT	≤ 0.20	98 (83.1)	20 (16.9)	**0.015**
	0.21–0.65	17 (60.7)	11 (39.3)	
	> 0.65	4 (57.1)	3 (42.9)	
	Missing 1			
Prognostic stage post-NACT	0	49 (96.1)	2 (3.9)	**0.009**
	IA	37 (71.2)	15 (28.8)	
	IB	5 (71.4)	2 (28.6)	
	IIA	9 (75.0)	3 (25.0)	
	IIB	4 (57.1)	3 (42.9)	
	IIIA	6 (54.5)	5 (45.5)	
	IIIB	10 (71.4)	4 (28.6)	
Metastasis	Yes	21 (65.6)	11 (34.4)	0.063
	No	98 (81.0)	23 (19.0)	
	Missing 1			
Proliferation index (Ki-67) pre-NACT	≤ 20%	7 (77.8)	2 (22.2)	0.981
	> 20%	113 (77.9)	32 (22.1)	
Breast Tumoral response	pCR	59 (93.7)	4 (6.3)	**0.001**
	pPR	61(67.0)	30 (33.0)	
Lymph nodes Response	pCR	85 (86.7)	13 (13.3)	**0.001**
	pPR	31(67.4)	15 (32.6)	
	pNR	4 (40.0)	6 (60.0)	
ER expression pre-NACT	< 1%	56 (90.3)	6 (9.7)	**0.002**
	≥ 1%	64 (69.6)	28 (30.4)	
PR expression pre-NACT	< 1%	80 (87.0)	12 (13.0)	**0.001**
	≥ 1%	40 (64.5)	22 (35.5)	
AR expression pre-NACT	< 10%	11 (78.6)	3 (21.4)	0.951
	≥ 10%	109 (77.9)	31 (22.1)	
Mortality	Death	15 (78.9)	4 (21.1)	0.870
	Alive	105 (77.8)	30 (22.2)	
RCT post-NACT	> 5%	46 (64.8)	25 (35.2)	**0.001**
	≤ 5%	73 (90.1)	8 (9.9)	
	Missing 2			
Proliferation index (Ki-67) post-NACT	≤ 20%	83 (83.0)	17 (17.0)	**0.039**
	> 20%	37 (67.5)	17 (31.5)	
TILs pre-NACT	< 10%	18 (78.3)	5 (21.7)	0.926
	≥ 10%	57 (79.2)	15 (20.8)	
	Missing 59			

Significant values are in bold.

SI Table 1 highlights no significant differences in tumor and lymph node response to neoadjuvant therapy between luminal and non-luminal HER2+ BC subtypes (P = 0.060 and P = 0.134, respectively). Moreover, non-luminal HER2+ BC showed higher prevalence of apocrine histotype (P = 0.012), high grade (G3; P = 0.001), higher post-NACT prognostic stage (P = 0.029), higher values of pre-NACT TILs (P = 0.002), less frequently HER2 IHC2+ (P = 0.002) and more frequently pre-NACT AR negativity (P = 0.013).

### Hormonal receptor modifications in HER2+ BC subtype treated with neoadjuvant therapy

The expression value of ER, PR and AR, as well as ki67, was measured in pre- and post-NACT condition in patients with pathological partial response (pPR). ER+ HER2+ BC subtype (ER ≥ 1%) at diagnosis, did not show a significant modification in ER expression caused by the therapy (P = 0.343). In PR+ HER2+ BC at diagnosis (PR ≥ 1%), a significant post-NACT reduction of the receptor expression was revealed (P = 0.002). AR+ HER2+ BC at diagnosis (AR ≥ 10%) decreased after neoadjuvant therapy (P = 0.050). No differences in pre- and post-NACT hormonal receptor expression have been observed in non-luminal HER2+ BC as it might be expected, due to anti-proliferative therapy effect, post-NACT ki67 expression underwent a significant reduction (P < 0.001) (SI Table 2).

### Tumor response to neoadjuvant therapy in HER2+ BC subtype

The tumoral pCR rate in HER2+ BC subtype was 40.9%. Clinico-pathological parameters in tumors with pCR vs those with pPR showed that pCR has been associated with pre-NACT G3 (P = 0.014), HER2 IHC3+ (P < 0.001), low LNR (≤ 0.20, P = 0.07), low post-NACT prognostic stage (stage 0; P < 0.001), low post-NACT ki67 (P < 0.001), low post-NACT RTC (P < 0.001), high lymph node response to NACT (P < 0.001), scarce distant metastasis (P = 0.005) and high survival (P = 0.017). Tumoral pCR has been more frequently observed in right breast diagnosis (P = 0.012) and showed higher TILs pre-NACT (P = 0.009) (Table 2). Multivariate analysis showed that HER2 IHC3+ and TILs ≥ 10% are independent predictive factors favoring pCR, contrarily left-sided tumors reduce the probability to obtain pCR (Table 3).

**Table 2:** Clinico-pathological data of 154 patients with HER2-positive breast cancer based on tumoral response to NACT.

		pCR (n = 63) *n (%)*	pPR (n = 91) *n (%)*	P-value
Age (year)	< 54	29 (39.2)	45 (60.8)	0.676
	≥ 54	34 (42.5)	46 (57.5)	
Histologic type pre-NACT	NST	56 (43.1)	74 (56.9)	0.409
	ILC	1 (11.1)	8 (88.9)	
	APOCRINE	3 (33.3)	6 (66.7)	
	MICROPAPILLARY	1 (50.0)	1 (50.0)	
	MUCINOUS	2 (50.0)	2 (50.0)	
Histologic grade pre-NACT	G2	5 (19.2)	21 (80.8)	**0.014**
	G3	58 (45.3)	70 (54.7)	
HER2 score pre-NACT	2+	4 (11.8)	30 (88.2)	**0.001**
	3+	59 (49.2)	61 (50.8)	
Tumor size (ypT)	ypT0	63 (100.0)	0 (0.0)	**0.001**
	ypT1a	0 (0.0)	13 (100.0)	
	ypT1b	0 (0.0)	13 (0.0)	
	ypT1c	0 (0.0)	20 (100.0)	
	ypT2	0 (0.0)	32 (100.0)	
	ypT3	0 (0.0)	6 (100.0)	
	ypT4	0 (0.0)	7 (100.0)	
Lymph node status (ypN)	ypN0	51 (52.0)	47 (48.0)	**0.001**
	ypN1	11 (31.4)	24 (68.6)	
	ypN2	0 (0.0)	14 (100.0)	
	ypN3	1 (14.3)	6 (85.7)	
Lymph node ratio Post-NACT	≤ 0.20	56 (47.5)	62 (52.5)	**0.007**
	0.21–0.65	7 (25.0)	51 (75.0)	
	> 0.65	0 (0.0)	7 (100.0)	
	Missing 1			
Prognostic stage Post-NACT	0	51 (100.0)	0 (0.0)	**0.001**
	IA	7 (13.5)	45 (86.5)	
	IB	0 (0.0)	7 (100.0)	
	IIA	3 (25.0)	9 (75.0)	
	IIB	0 (0.0)	7 (100.0)	
	IIIA	0 (0.0)	11 (100.0)	
	IIIB	2 (14.3)	12 (85.7)	
Metastasis	Yes	6 (18.8)	26 (81.3)	**0.005**
	No	56 (46.3)	65 (53.7)	
	Missing 1			
Proliferation index (Ki-67) pre-NACT	≤ 20%	3 (33.3)	6 (66.7)	0.634
	> 20%	60 (41.4)	85 (58.6)	
Lymph nodes Response	pCR	51 (52.0)	47 (48.0)	**0.001**
	pPR	12 (26.1)	34 (73.9)	
	pNR	0 (0.0)	10 (100.0)	
ER expression pre-NACT	< 1%	31 (50.0)	31 (50.0)	0.060
	≥ 1%	32 (34.8)	60 (65.2)	
PR expression pre-NACT	< 1%	44 (47.8)	48 (52.2)	**0.033**
	≥ 1%	19 (30.6)	43 (69.4)	
AR expression pre-NACT	< 10%	5 (35.7)	9 (64.3)	0.678
	≥ 10%	58 (41.4)	82 (58.6)	
Mortality	Death	3 (15.8)	16 (84.2)	**0.017**
	Alive	60 (44.4)	75 (55.6)	
SITE	Right	40 (50.6)	39 (49.4)	**0.012**
	Left	23 (30.7)	52 (69.3)	
TILs pre-NACT	< 10%	5 (21.7)	18 (78.3)	**0.009**
	≥ 10%	38 (52.8)	34 (47.2)	
	Missing 59			
RTC post-NACT	> 5%	0 (0.0)	71 (100.0)	**0.001**
	≤ 5%	63 (77.8)	18 (22.2)	
	Missing 2			
Proliferation index (Ki-67) Post NACT	≥ 20%	59 (59.0)	41 (41.0)	**0.001**
	> 20%	4 (7.4)	50 (92.6)	

Significant values are in bold.

**Table 3:** Logistic regression to assess the relationship between pCR or pPR and clinicopathological features in HER2+ breast cancer patients.

	Univariate analysis		Multivariate analysis	
	OR (95% CI)	P value	OR (95% CI)	P value
HER2 score (IHC 2+ vs IHC 3+)	0.14 (0.04–0.38)	0.001	0.10 (0.02–0.38)	0.002
Histologic grade pre-NACT (G2 vs G3)	0.29 (0.09–0.76)	0.018	0.65 (0.12–3.00)	0.596
TILs pre-NACT (< 10% vs ≥ 10%)	0.25 (0.08–0.70)	0.013	0.17 (0.04–0.61)	0.009
Site (right vs left)	2.32 (1.21–4.53)	0.012	3.94 (1.51–11.16)	0.007
ER expression pre-NACT (< 1% vs ≥ 1%))	1.87 (0.97–3.64)	0.061	0.75 (0.26–2.10)	0.592

### Laterality and tumor response in HER2+ BC subtype treated with neoadjuvant therapy

A higher frequency of pCR in the right breast cancer compared to the left one was observed, 63.5% of pCR occurring in the right breast vs 36.5% in the left (P = 0.012). No differences were observed in the distribution of HER2 values (IHC 3+, IHC 2+; P = 0.575) and in tumor grade (G2, G3) between the right and left breasts (P = 0.151). Nevertheless, pre- and post-NACT ki67 expression was not significantly different between left and right breasts (P = 0.342 and P = 0.565, respectively). While, lower distant metastasis (P < 0.001) and mortality (P = 0.020) were observed in the right-sided BC compared to left. (SI Table 3).We found a higher frequency of RTC in the left breast (> 5%, P < 0.001) (SI Table 3). A higher complete response frequency with residual cellularity equal to 0 in the right breast is observed: this could cause an unbalance in this observation. To better evaluate this finding, we repeated the RTC/site measurement only on pPR patients where a residual tumor was present. We confirm that the RTC frequency in the left breast is higher compared to the right one (> 5%, P < 0.001). This scenario pointed out that, even in the case of a tumoral pPR, a lower RTC is observed in the right breast (Table 4).

**Table 4:** Clinico-pathological data of 91 HER2-positive breast cancer patients with pathological partial response based on tumor localization.

		Right (n = 39)	Left (n = 52)	P value
Age (year)	< 54	18 (40.0)	27 (60.0)	0.3586
	> 54	21 (45.7)	25 (54.3)	
Histologic type pre-NACT	NST	32 (43.2)	42 (56.8)	0.792
	ILC	3 (37.5)	5 (62.5)	
	APOCRINE	2 (33.3)	4 (66.7)	
	MICROPAPILLARY	1 (100.0)	0 (0.0)	
	MUCINOUS	1 (50.0)	1 (50.0)	
Histologic grade pre-NACT	G2	6 (28.6)	15 (71.4)	0.131
	G3	33 (47.1)	37 (52.9)	
Tumor size (ypT)	ypT0	0 (0.0)	0 (0.0)	**0.008**
	ypT1a	11 (84.6)	2 (15.4)	
	ypT1b	7 (53.8)	6 (46.2)	
	ypT1c	7 (35.0)	13 (65.0)	
	ypT2	9 (28.1)	23 (71.9)	
	ypT3	1 (16.7)	5 (83.3)	
	ypT4	4 (57.1)	3 (42.9)	
Lymph node status (ypN)	ypN0	24 (51.1)	23 (48.9)	0.245
	ypN1	10 (41.7)	14 (14.3)	
	ypN2	4 (28.6)	10 (71.4)	
	ypN3	1 (16.7)	5 (83.3)	
Lymph node ratio post-NACT	≤ 0.20	31 (50.0)	31 (50.0)	0.112
	0.21–0.65	7 (33.3)	14 (66.7)	
	> 0.65	1 (14.3)	6 (85.7)	
	Missing 1			
Prognostic stage post-NACT	0	0 (0.0)	0 (0.0)	**0.042**
	IA	26 (57.8)	19 (42.2)	
	IB	1 (14.3)	6 (85.7)	
	IIA	2 (22.2)	7 (77.8)	
	IIB	3 (42.9)	4 (57.1)	
	IIIA	2 (18.2)	9 (81.8)	
	IIIB	5 (41.7)	7 (58.3)	
Metastasis	Yes	9 (34.6)	17 (65.4)	0.315
	No	30 (46.2)	35 (53.8)	
Proliferation index (Ki-67) pre-NACT	≤ 20%	3 (50.0)	3 (50.0)	0.715
	> 20%	36 (42.4)	49 (57.6)	
Lymph nodes Response	pCR	24 (51.1)	23 (48.9)	0.155
	pPR	13 (38.2)	21 (61.8)	
	pNR	2 (20.0)	8 (80.0)	
ER expression pre-NACT	< 1%	15 (48.4)	16 (51.6)	0.444
	≥ 1%	24 (40.0)	36 (60.0)	
PR expression pre-NACT	< 1%	19 (39.6)	29 (60.4)	0.505
	≥ 1%	20 (46.5)	23 (53.5)	
AR expression pre-NACT	< 10%	5 (55.6)	4 (44.4)	0.417
	≥ 10%	34 (41.5)	48 (58.5)	
Mortality	Death	5 (31.3)	11 (68.8)	0.301
	Alive	34 (45.3)	41 (54.7)	
RCT post-NACT	> 5%	23 (32.4)	48 (67.6)	**0.001**
	≤ 5%	16 (88.9)	2 (11.1)	
	Missing 2			
HER2 Score pre-NACT	2+	14 (46.7)	16 (53.3)	0.607
	3+	25 (41.0)	36 (59.0)	
TILS pre-NACT	< 10%	8 (44.4)	10 (55.6)	0.664
	≥ 10%	13 (38.2)	21 (61.8)	
	Missing 39			
Proliferation index (Ki-67) post-NACT	≤ 20%	17 (41.5)	24 (58.5)	0.808
	> 20%	22 (44.0)	28 (56.0)	

Significant values are in bold.

### TILs value as a predictor of pathologic complete response in breast parenchyma

Evaluating TILs value as a continuous variable, there was no significant difference in pre-NACT TILs in laterality and luminal/non-luminal subtypes. Immune cells were more represented in the pre-NACT G3 compared to G2 (P = 0.046). The tumoral pCR group had a higher percentage of pre-NACT infiltrating immune cells compared to the pPR group (P = 0.003), while pre-NACT TILs negatively correlate with residual cellularity after neoadjuvant therapy (P = 0.004) (Table 5).

**Table 5:** Comparison of Pre-NACT TILs between different groups of clinico-pathological parameters. A Shapiro–Wilk test showed that the distribution of TILs departed significantly from normality (W = 0.896, P-value < 0.01). Mann–Whitney test was used, and P value was significant at 0.05.

Variables	Mean	Median	P value
Left	28.3	15.0	0.331
Right	34.4	25.0	
Pre-NACT G2	18.5	5.0	0.046
Pre-NACT G3	33.5	22.5	
Pre-NACT Luminal HER2	28.0	15.0	0.053
Pre-NACT non-Luminal HER2	36.6	30.0	
Pathological tumor response (pCR)	40.1	30.0	0.003
Pathological tumor response (pPR)	24.3	15.0	
RTC > 5%	22.8	15.0	0.004
RTC ≤ 5%	38.5	30.0	

### Machine learning in the prediction of parenchymal tumor response

The results of the classification models capable of predicting tumor response (complete or partial) in BC have shown an overall accuracy of nearly 80%, with sensitivity and specificity rates both in the order of 0.80 (SI Table 4). These early assessments can be considered promising given the inherent difficulty of inducing prediction models from small sets of training instances. The AUC scores, higher than 0.8, reveal the presence of rather strong, non-random patterns, despite the suboptimal dimensionality of the dataset, and deserve to be fully explored by further experiments on larger datasets. We evaluated the effectiveness of each algorithm based on patient characteristics (age, location, histotype, grade, ER, PR, AR, KI67, percentage of HER2-positive cells, and HER2-score). The k-NN classifier (with k = 5) proved to be the most accurate in our machine learning experiments (SI Table 4). The two selection methods that gave the best results were: (i) OneR, a univariate selection approach that evaluates the strength of every single feature independently of the others, and (ii) ReliefF, a multivariate approach that also takes interdependencies among features into account. Both methods correlate by ranking the pre-NACT HER2 score and BC laterality in the first two positions, as shown in Table 6. Using these two attributes the k-NN classifier achieves a noteworthy overall accuracy of 71.3%, compared to 79.1% (k = 5) obtained with all attributes, with a sensitivity value of 0.59, a specificity value of 0.82 and an AUC value of 0.71. Using the first five attributes selected by ReliefF, an overall accuracy of 76.7% is obtained, with sensitivity and specificity of 0.74 and 0.79 respectively, and an interesting AUC value of 0.81. It would seem that, in addition to HER2 score and BC laterality, the pre-NACT ER and AR attributes are also relevant in contributing to a better performance on the prediction model. In particular, AR ranks well only in the multivariate ranking produced by ReliefF and it appears to play a role in improving overall results.

**Table 6:** Attribute ranking from two different Feature selection methods: Univariate OneR and Multivariate RelieF.

OneR		RelieF	
Attribute	Rank	Attribute	Rank
Pre NACT HER2 Score	1	Pre NACT HER2 Score	1
Breast laterality	2	Breast laterality	2
Pre NACT HER2 Cell %	3	Pre NACT ER	3
Pre NACT Grade	4	Pre NACT AR	4
Pre NACT ER	5	Pre NACT HER2 Cell %	5
Pre NACT PR	6	Pre NACT Grade	6
Pre NACT AR	7	Histotype	7
Histotype	8	Pre NACT PR	8
Pre NACT Ki67	9	Age	9
Age	10	Pre NACT Ki67	10

### Correlations of RTC with other clinico-pathological features of HER2+ BC subtype

A positive significant correlation was evident between RTC and the number of positive lymph nodes (P < 0.001). The RTC value also positively correlates with post-NACT ki67 (P < 0.001) giving an indication of the degree of response to therapy in terms of proliferative fraction. The post-NACT ER, PR and AR values were all positively correlated to RTC (all P < 0.001) (SI Table 5). This is consistent since luminal subtypes show less frequently a HER2 3+ over a HER 2+ amplified profile (P = 0.003) as well as a more frequent G2 over a G3 profile (P = 0.001) (SI Table 1). They are less responsive to NACT resulting in higher values of post-NACT RTC. Inverse correlations were observed between pre-NACT TILs and RTC (P = 0.003), and between pre-NACT TILs and post-NACT hormonal receptors (ER, P = 0.005; PR, P = 0.017, AR, P = 0.037). Metastatic lymph nodes were positively correlated with post-NACT ki67 (P < 0.001) and post-NACT hormonal receptors (ER, P = 0.002; PR, P = 0.008, AR, P = 0.005) (SI Table 5).

### Prognostic factors influencing outcome of HER2+ BC subtype treated with neoadjuvant therapy

**Figure 1:**
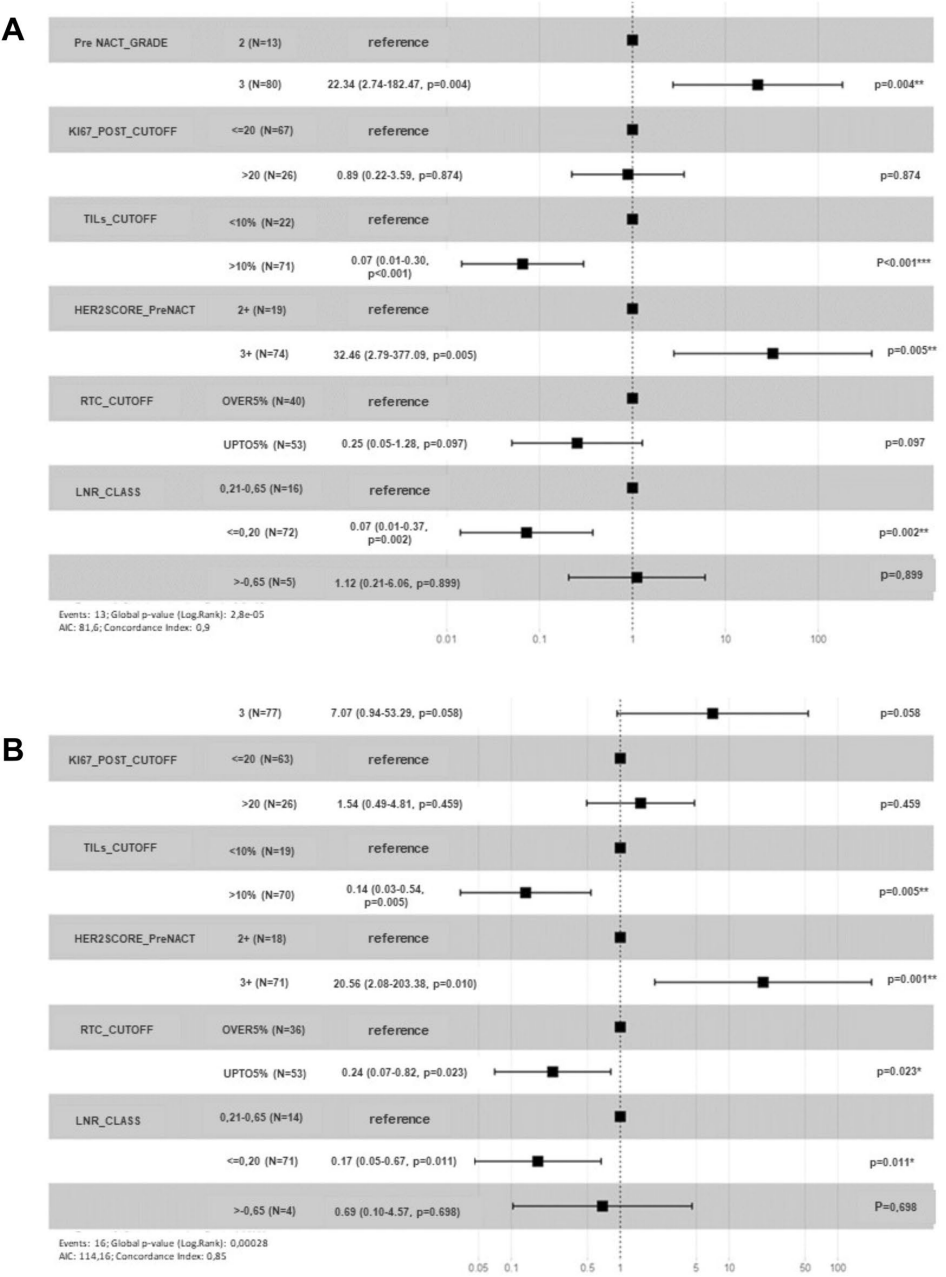
Multivariate analysis. (**A**) For disease-free survival in 154 patients with HER2+ breast cancer subtype treated with neoadjuvant therapy; (**B**) For overall survival in 154 patients with HER2+ breast cancer subtype treated with neoadjuvant therapy.

Multivariate analysis showed the following independent prognostic factors, RTC (0.24 CI 95% 0.07–0.82, P = 0.023), HER2 IHC3+ (20.56 CI 95% 2.08–203.38, P = 0.010), pre-NACT TILs (0.14 CI 95% 0.03–0.54, P = 0.005) and LNR (0.17 CI 95% 0.05–0.67, P = 0.011) (Fig. 1A). The Kaplan–Meier curve showed that the best DFS rate was found for HER2+ BC patients with ypT0-ypT1, ypN0, post-NACT stage 0–I (P = 0.013, P = 0.048 and P = 0.016, respectively), and with low RTC, tumoral pCR, lymph nodal pCR, low lymph node ratio, pre-NACT AR positivity, low post-NACT ki67 expression, high pre-NACT TILs and more frequently right-sided (P = 0.00031, P = 0.0041, P = 0.020, P = 0.00036, P = 0.011, P = 0.00072, P = 0.038 and P = 0.042, respectively) (SI Fig. 1).

**Figure 2:**
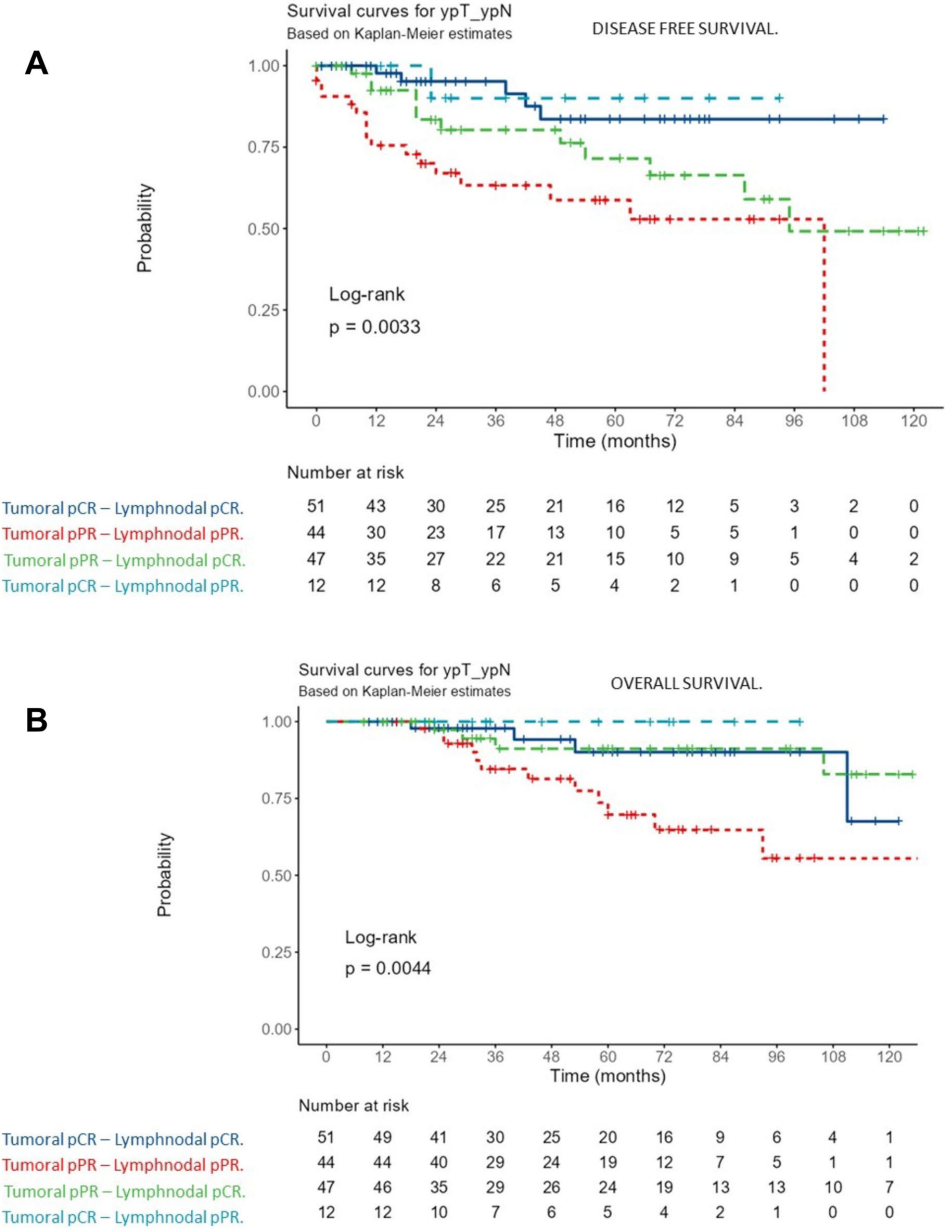
Kaplan–Meir curves. (**A**) For disease-free survival according to tumoral and lymph nodal response to NACT; (**B**) For overall survival according to tumoral and lymph nodal response to NACT.

The last update of the public registry revealed that 21 out of 154 (13.63%) of our patients died for disease related causes. The median OS was 40 months (SD± 33.1; range 116 months). Multivariate analysis showed that pre-NACT grade (OR 22.34, CI 95% 2.74–182.47 P = 0.004), pre-NACT TILs (OR 0.07, CI 95% 0.01–0.30 P < 0.001), HER2 expression (OR 32.46, CI 95% 2.79–377.09 P = 0.005) and LNR (OR 0.07, CI 95% 0.01–0.37 P = 0.002) are independent prognostic factors for OS (Fig. 1B). The Kaplan–Meier curve showed the best OS rate was found for ypN0 and post-NACT stage 0–I (P = 0.006, and P = 0.018, respectively) (SI Fig. 2). Moreover, a more favorable OS prognosis was associated with low RTC, lymph nodal pCR, low LNR, and high pre-NACT TILs (P = 0.044, P = 0.004, P = 0.002, P = 0.004, respectively) (Supplementary Fig. S2). The best DFS and OS were observed in patients with tumoral pCR independently of the complete or partial lymph node response to NACT (Fig. 2A,B).

## Discussion

Neoadjuvant approach plays a key role in clinical management of BC with unfavorable prognostic factors. The main purpose of NACT is to reduce tumor burden thus allowing surgery escalation^12^. Women with early-stage HER2+ BC (including stage I or IIA) are suitable candidates for neoadjuvant chemotherapy, if conservative surgery is not feasible for instance due to a high tumor-to-breast ratio^13^. In HER2+ BC subtype, a more aggressive tumor, neoadjuvant chemotherapy is strongly encouraged due to particularly sensitive to treatment and indication for adjuvant treatment too^6,7^. In HER2+ BCs, neoadjuvant therapy allows an early assessment of the efficacy of chemotherapy, the presence or absence of invasive disease after neoadjuvant therapy represents a prognostic and recalibration factor for subsequent therapies, i.e. following surgery^14^.

The HER2 positivity is associated with aggressive BC subtypes with poor outcome, and represents the marker to select patients for anti-HER2 therapies.

Our results identified predictive factors for pCR in HER2+ BC subtype across different HER2+ categories, revealing that HER2 IHC 3+ tumors had a more frequent tumoral and lymph nodal pCR, 93.7% and 86.7% respectively, compared to IHC 2+/SISH+. Supporting our data, HER2 IHC 3+ tumors showed more frequently yT0 and yN0, lower LNR, prognostic stage, ki67 expression and RTC after NACT, suggesting that the amount of HER2 protein overexpression on the cell surface regulates the efficacy of neoadjuvant therapy in HER2+ BC patients. Perez et al. investigated the efficacy of trastuzumab plus adjuvant chemotherapies, in a large randomized phase III clinical trial, showing that in patients with HER2 IHC−/FISH+ BC no advance in DFS has been obtained adding trastuzumab, while patients with HER2 IHC3+/FISH− BC exhibited DFS similar to those with HER2 IHC3+/FISH+ tumors, suggesting a key role of HER2 protein overexpression^15^. In our study HER2 IHC3+ BC, more responsive to NACT, is more frequently ER-/PR-. This agrees with the evidence that HER2+/ER− BC are likely to be highly dependent on the HER2 gene for growth, and typically show a good response to anti-HER2 therapies^16^. HER2+/ER+/PR+/AR+ BCs having pPR did not show variations in ER expression after NACT, while they showed PR and AR decrease associated with lower ki67 expression. This could probably depend on real loss expression after NACT rather than tumor heterogeneity. Persistent ER positivity could be the result of the prevalence of hormonal pathways in promoting tumor growth over HER2 and should explain the partial response to NACT. These findings highlight the value of considering combined HER2 and ER status to select patients who are most likely to benefit from neoadjuvant anti-HER2 therapy. Furthermore, our results showed no significant differences in tumor and lymph node response to NACT between luminal and non-luminal HER2+ BC. The predictive value of ER status for pCR after NACT has been very controversial^17–22^.

In the present study, NACT therapy was helpful in achieving a pCR rate of 40.9%, which is in line with previous reports showing a pCR ranging from 34 to 76%^17,19,21,23,24^. Patients with pCR have the best survival with a mortality rate of about 15.8% vs 84.2% of those with pPR.

Our data highlight that HER2 IHC3+ is an independent predictive factor of pCR after NACT, in keeping with previous reports^23,25,26^. We identify other two independent predictive factors of pCR in HER2+ BC subtype: the right-sided diagnosis and the amount of TILs. Our data did not show a difference of BC incidence between right-sided and left-sided. They also showed a higher frequency of pCR in the right breast compared to the left one, with right-sided tumors showing very low distant metastasis and increased survival. Considering that different lymphatic flux was demonstrated between the right and left side of breast^27^ it may be assumed that neoplastic cells, which pass through the left lymphatic flow, are more likely to pass into the systemic circulation due to rapid flow, inducing an increased risk of earlier distant metastases compared to right BC. Karats et al. have demonstrated that left laterality in BC patients is an independent prognostic factor related with shorter time to first metastasis, increased risk of distant metastases. Abdou et al. detected that left-sided BC have a more proliferative genomic profile, although are less responsive to neoadjuvant chemotherapy, and getting worse long-term outcomes compared to right-sided BC^28^. We demonstrated that the abundance of immune cells prior to neoadjuvant was significantly associated with more aggressive clinico-pathological parameters, as G3 and ER negativity in HER2+ BC. Hwang et al. identified that higher pre-NACT TILs were correlated with more aggressive clinicopathological features as higher cT staging, histological grade, and Ki-67 index^29^.

Although, the effects of TILs on neoadjuvant trastuzumab plus chemotherapy on HER2+ BC patients remain controversial^30–32^, our data showed that a higher percentage of infiltrating immune cells was present in tumor pCR group compared to pPR group and associated with low RTC post-NACT levels. This data validates pre-NACT TILs as a persistent biomarker for response to current anti-HER2 treatment. The activation of PI3K/AKT/NF-kB pathways, downstream to HER2 oncogene, stimulates the tumor cell production of CCL2^33^. CCL2 induces recruitment and activation of TILs at the tumor site eventually causing an improved trastuzumab-immune-mediated anti-tumor activity. Then, the highest HER2 protein overexpression in HER2+ BC was related to a major immune-responsiveness and consequently higher pCR. These results propose that TILs determination should be considered for HER2+ BC patient stratification and follow up.

In this work, we took advantage of machine learning techniques by performing a so-called supervised approach, namely classification. We compared several classification algorithms and chose the most accurate one for the dataset being studied. We created a model capable of predicting the occurrence of the tumor response event in breast cancers, whether partial or complete, with a measurable degree of accuracy through a ten-fold cross-validation approach. Furthermore, by applying some of the most widely used evaluation metrics (accuracy, sensitivity, specificity, and AUC), we have shown that even with such a small dataset and a small number of variables, our induced model reveals non-random patterns in predicting neoadjuvant outcomes. In this analysis context, we have also used some feature selection techniques to dig into the variable's predictive power and find the most informative subset of features for our classification task. Results indicate that although the considered variables are not able to encompass the entire clinical history of the patient, they are still highly informative for the purposes of response to the neoadjuvant therapy occurring in the breast. Two variables strongly emerge from different feature selection techniques as valid predictors of tumor response: HER2 IHC score and laterality. While the role of the pre-NACT HER2 score, here confirmed by a machine learning approach, has been reported many times, to our knowledge this is the first time that the role of laterality emerges from a machine learning analysis. Abdou et al^.^ recently reported left-sided tumors with lower pCR rates versus right-sided tumors^28^. Here we confirm and extend this observation with a different approach, on a comparably sized dataset focused on HER2 BC subtype. We are confident that such an approach as machine learning, coupled with more classical analytical methods, especially applied on larger datasets, could be of great use in extending the meaning of biomarkers valuable at predicting the outcome of neoadjuvant therapy.

Concerning the prognostic significance of the pathological parameters analyzed, we showed that HER2 IHC3+ score acts as an unfavorable prognostic factor in HER2+ BC patients, as does higher histologic grade. Otherwise, higher pre-NACT TILs, lower LNR and lower RTC behave as good prognostic factors for the same patients. In particular, we confirmed the prognostic significance of tumoral pCR as a biomarker to increase DFS and OS in HER2+ BC, independently of the lymph nodal response to NACT, as well as low post-NACT ki-67 expression and right-sided tumor. In our study, patients having a measured RTC ≤ 5% had on average longer survival and a better DFS than those with a measured cellularity > 5%. Multivariate analysis also showed that higher RTC (> 5% vs ≤ 5%) was associated with increased risk of progression (P = 0.023). These findings support the use of RTC within the tumor as an informative parameter that could be integrated in the pathologic assessment of HER2 BC undergoing NACT. These results support recent knowledge on the prognostic role of residual cellularity in tumors treated with neoadjuvant therapy^34,35^.

Our study does have some limitations that are primarily focused on its retrospective strategy. Hence, some information on clinical follow-up data were not completely included in the medical records. Additionally, the analysis should be expanded to more patients in the coming years to consolidate and reproduce our results.

Our study emphasizes the significant role of the HER2 protein expression levels for NACT response in HER2+ BC subtype. We demonstrated that HER2 IHC3+ is a good predictive factor of pCR, although they represent unfavorable prognostic factors for DFS and OS in patients with HER2+ BC subtype. The same biomarker could be utilized in the application of neoadjuvant therapeutic options of the HER2+ BC patients but might be considered during decision-make follow-up in the same patients. Other two independent predictive factors of pCR in HER2+ BC subtype as the right-sided and TILs have been demonstrated. Furthermore, we also showed that increased pre-NACT TILs concentrations with a meaningful cut-off value of 10%, lower LNR and lower RTC, below 5%, are associated with better outcome in HER2+ BC, suggesting that the immune microenvironment and scarce lymph node involvement have a crucial role in a patient's prognosis. The combination of all predictors described might offer new options for the prediction of NACT effectiveness and for the stratification of HER2+ BC allowing different therapeutic strategies during the clinical decision making.

## Methods

A review of surgical specimens from the archives of the Department of Pathology, Oncology Hospital, Cagliari, Italy, identified a retrospective cohort of 154 women diagnosed with invasive HER2+ BC and treated with neoadjuvant systemic therapy followed by surgical resection between October 2010 and May 2021. All patients received trastuzumab-based neoadjuvant chemotherapy (NACT): four cycles of doxorubicin plus cyclophosphamide every 2–3 weeks and sequential administration of weekly paclitaxel 80 mg/m^2^ given for 12 consecutive doses or docetaxel 100 mg/m^2^ every 3 weeks and concomitant trastuzumab for 12 weeks. All patients completed the neoadjuvant therapy regimen before surgery. We excluded patients with bilateral BC or other malignancies. All eligible patients were confirmed to be diagnosed with invasive HER2+ BC by core needle biopsy. Three experienced pathologists independently reviewed all cases. The study protocol was approved by the local research ethics committee (File number PG/2021/14264) following the Italian guidelines for conducting retrospective observational studies (G.U. n. 76, 31/03/2008). Only coded data were collected to save patient confidentiality.

### Immunohistochemistry

The IHC analysis was performed utilizing specific antibodies against ER Clone SP1 (Ventana Medical Systems Inc., Tucson, AZ, USA); PR Clone 1E2 (Ventana Medical Systems); AR Clone SP107 (Cell-MarqueTM, Rocklin, CA, USA); HER2 PATHWAY Clone 4B5 (Ventana Medical Systems); Ki67 Clone 30-9 (Roche Diagnostics K.K., Tokyo, Japan). We used the Ventana Benchmark XT staining system with Optiview DAB detection kit for immunostaining on 3 µm-thick tissue sections of FFPE specimens. HER2 gene amplification by ultra-View SISH Detection Kit (Ventana Medical Systems) was performed in cases of HER2 with equivocal immunohistochemical score (IHC 2+). Evaluation of immunostaining and dual-probe in situ hybridization for HER2 was based on American Society of Clinical Oncology/College of American Pathologists (ASCO/CAP) recommendations^1^. ER and PR expression were assessed positive if at least 1% immunostained tumor nuclei were detected in the sample, according to ASCO/CAP recommendations for immunohistochemical testing of hormone receptors in BC^36^. The Ki67 cut-off was ≤ 20% and > 20%^37^. AR expression was considered positive if at least 10% immunostained tumor nuclei were detected^38^. All IHC expressions were assessed using a semi-quantitative method.

### Baseline data

Demographic and clinical data were retrieved from medical records. Histologic types were determined according to the UICC-WHO criteria^39^ and tumor grade was established according to the Nottingham scheme^40^. Prognostic stage was defined according to the 8th edition of the American Joint Committee on Cancer criteria (AJCC)^41^. Lymph node ratio (LNR) was defined as the number of positive lymph nodes divided by the number of evaluated lymph nodes (cut-off points were < 0.21, 0.21–0.65, and > 0.65)^42^. Post-NACT RTC was assessed by reviewing hematoxylin and eosin (HE) slides obtained after surgery. RTC was assessed by comparing tumor cellularity before and after treatment and defined as the percentage of residual invasive tumor cells^43^. The RTC cut-off was established as ≤ 5% and > 5%. ER, PR, AR, Ki67, HER2 expression were assessed before and after neoadjuvant treatment when residual tumor was present. The pre and post NACT Ki67 expressions were calculated. A pre-NACT TILs assessment was performed. TILs was defined as the overall percentage of the stromal area within the borders of the invasive tumor that is covered by mononuclear immune cells^44^. TILs cut off of 10% was established consistently with Kurozumi et al.^45^. Overall survival (OS) was defined as the time from diagnosis to death or last follow-up; disease-free survival (DFS) was the time between post-NACT surgery and first recurrence. Tumoral pCR was defined as the absence of invasive cancer in the breast irrespective of remaining DCIS in the primary tumor (ypT0/is). Lymph nodal pCR was defined as the absence of positive lymph nodes (ypN0)^8^.

### Machine learning

We leveraged some machine learning techniques to predict tumor response in breast cancer. We tested the following algorithms: k-Nearest Neighbor (k-NN), Simple Logistic (SL), Support Vector Machine (SVM), Multi-Layer Perceptron (MLP), Random Forest (RF). All the experiments were conducted with a tenfold cross-validation approach^46,47^. To globally evaluate the resulting performance, we leveraged some well-known metrics: overall percentage of correct predictions (accuracy), fraction of correctly classified positive instances (sensitivity), fraction of correctly classified negative instances (specificity), and the area under the ROC Curve (AUC), which provides a single score to summarize the classifier performance at different probability thresholds for the positive class^46^. The latter gives us a complete response in breast cancer. To assess the extent to which a feature or feature subset contributes to the quality of the prediction, we used feature selection techniques that exploit algorithms and heuristics suitable for assessing the strength of the correlation between the input attributes and the target class^48^, thus allowing the identification of irrelevant or noisy factors. We considered two feature selection methods that worked best: OneR^48,49^, a univariate selection approach that evaluates the strength of each individual feature independently of the others, and ReliefF^48,49^, a multivariate approach that also takes interdependencies among features into account. For the machine learning tasks involved in our study, we chose the WEKA machine learning workbench, as it provides a large collection of algorithms as well as a rich set of supporting functions (https://www.cs.waikato.ac.nz/ml/weka/). Indeed, it contains tools for data preprocessing, classification, regression, clustering, association rules mining, feature selection, and visualization. The software is released under the GNU General Public License, thus ensuring reproducibility and consistency.

### Statistical analysis

Quantitative variables were described by median and interquartile range (IQR) according to non-normal distribution, whereas absolute and relative (percentages) frequencies were used for qualitative variables. Statistical differences for qualitative variables were evaluated using Chi2 or Fisher’s exact tests, when appropriate. Wilcoxon test and Mann–Whitney were used on continuous variables when appropriate. Also Spearman's Rho metric for continuous variables was used when normality conditions are not met. A Kaplan–Meier curve and Log-Rank test was performed to describe OS and DFS according to clinico-pathological and molecular variables. Multivariate Cox regression analysis was performed to evaluate the association between OS, DFS and clinico-pathological features, and molecular variables. The statistical significance was set up at P = 0.05. Statistical analysis was carried out using STATA®16 (StataCorp, College Station, TX, USA).

### Ethics approval and informed consent

The study was conducted in accordance with the Declaration of Helsinki and approved by the ethics committee “Azienda Ospedaliero Universitaria” Cagliari, Italy (File number PG/2021/14264). For this study informed consent has been waived by “Azienda Ospedaliero Universitaria” ethics committee due to the anonymity and retrospective nature of the study.

### Supplementary Information

Supplementary Figure S1.

Supplementary Figure S2.

Supplementary Table S1.

Supplementary Table S2.

Supplementary Table S3.

Supplementary Table S4.

Supplementary Table S5.

## Data Availability

The data that support the findings of this study are available from the corresponding author upon reasonable request.
