# Supplementary figures and images for: Biomarker dynamics affecting neoadjuvant therapy response and outcome of HER2-positive breast cancer subtype
Source: Sci Rep. 2023 Aug 8;13:12869. doi: 10.1038/s41598-023-40071-2 (PMC10409859; doi:10.1038/s41598-023-40071-2)

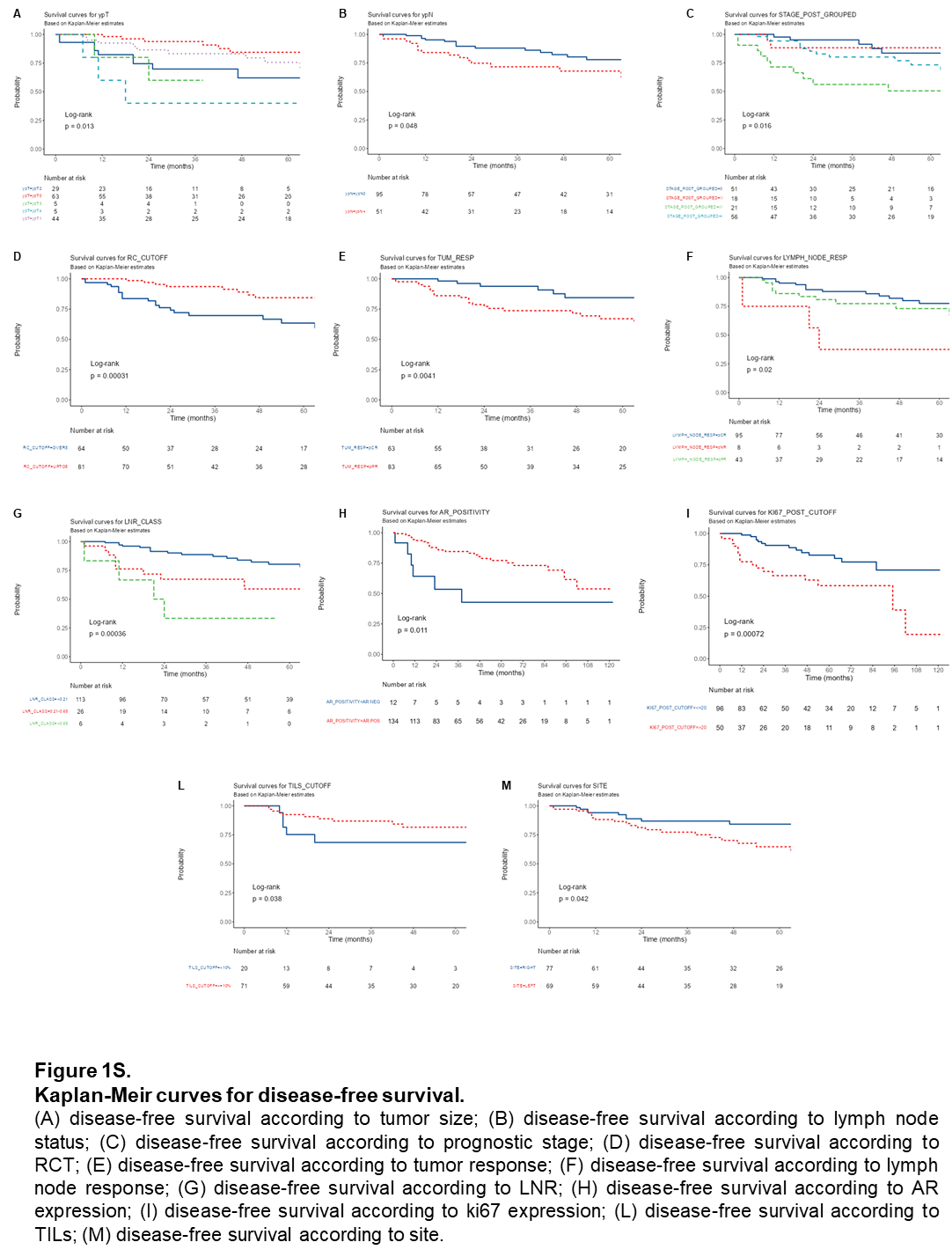

Supplement: Supplementary file 1 — Supplementary Figure S1. [file 41598_2023_40071_MOESM1_ESM.docx]

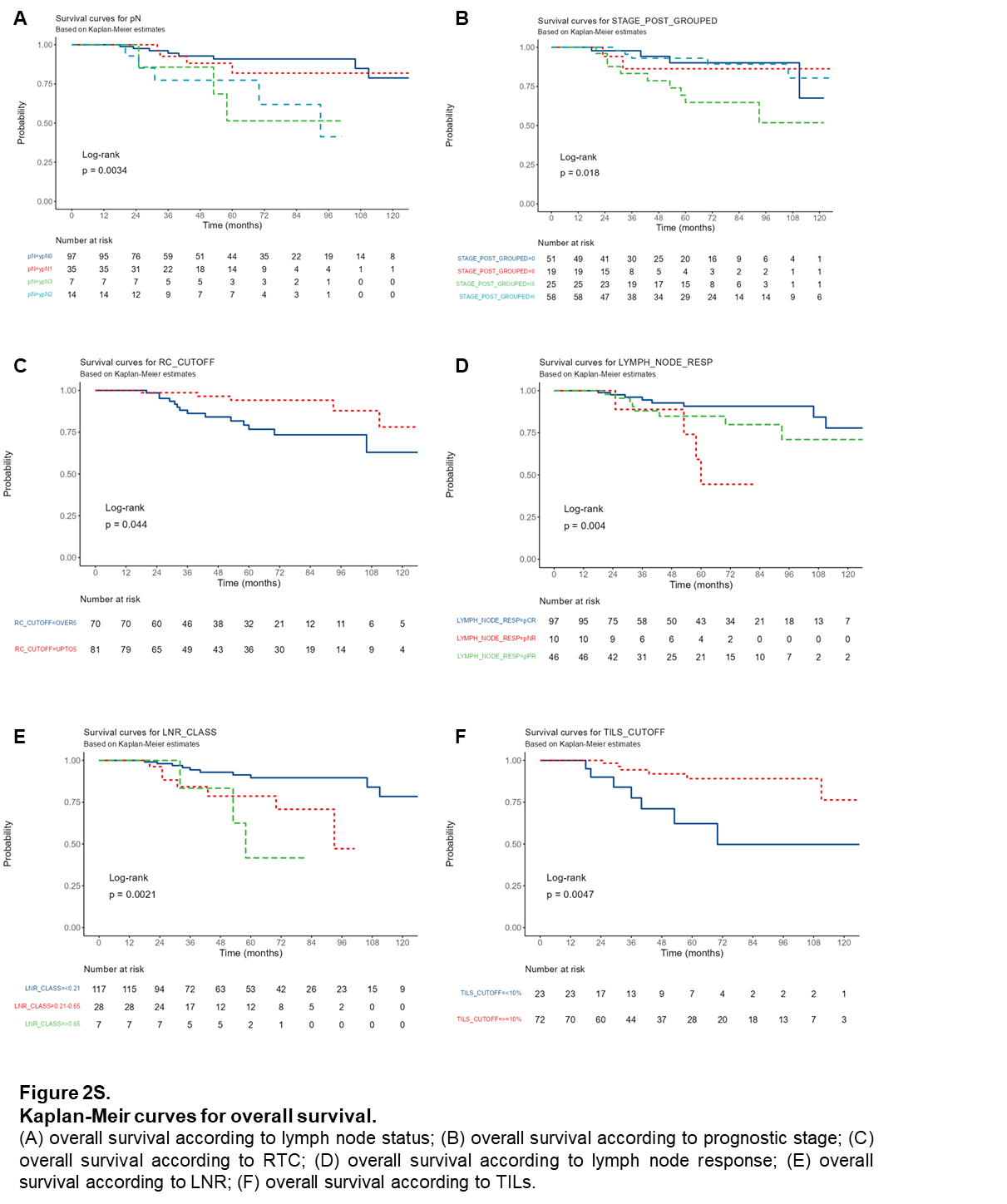

Supplement: Supplementary file 2 — Supplementary Figure S2. [file 41598_2023_40071_MOESM2_ESM.docx]
