# Supplementary material for: Biomarker dynamics affecting neoadjuvant therapy response and outcome of HER2-positive breast cancer subtype
Source: Sci Rep. 2023 Aug 8;13:12869. doi: 10.1038/s41598-023-40071-2 (PMC10409859; doi:10.1038/s41598-023-40071-2)
Supplement: Supplementary file 3 — Supplementary Table S1. [file 41598_2023_40071_MOESM3_ESM.docx]

**Supplementary Table S1. Clinico-pathological data of 154 patients with HER2-positive breast cancer based on ER expression**

|  | | **LUMINAL HER2 (n=92) n (%)** | **NON-LUMINAL HER2 (n= 62) n (%)** | **p-value** |
| --- | --- | --- | --- | --- |
| ***Age (year)*** | *<54* | 49 (66.2) | 25 (33.8) | 0.115 |
|  | *≥54* | 43 (53.8) | 37 (46.3) |  |
|  |  |  |  |  |
| ***Site*** | *RIGHT* | 48 (60.8) | 31 (39.2) | 0.791 |
|  | *LEFT* | 44 (58.7) | 31 (41.3) |  |
|  | |  |  |  |
| ***Histologic type pre-NACT*** | *NST* | 79 (60.8) | 51 (39.2) | **0.012** |
|  | *ILC* | 7 (77.8) | 2 (22.2) |  |
|  | *APOCRINE* | 1 (11.1) | 8 (88.9) |  |
|  | *MICROPAPILLARY* | 1 (50.0) | 1 (50.0) |  |
|  | *MUCINOUS* | 4 (100.0) | 0 (0.0) |  |
|  |  |  |  |  |
|  | |  |  |  |
| ***Histologic grade pre-NACT*** | *G2* | 23 (88.5) | 3 (11.5) | **0.001** |
|  | *G3* | 69 (53.9) | 59 (46.1) |  |
|  | |  |  |  |
| ***Tumor size (ypT)*** | *ypT0* | 32 (50.8) | 31 (49.2) | 0.452 |
|  | *ypT1a* | 9 (69.2) | 4 (30.8) |  |
|  | *ypT1b* | 9 (69.2) | 4 (39.8) |  |
|  | *ypT1c* | 11 (55.0) | 9 (45.0) |  |
|  | *ypT2* | 22 (68.8) | 10 (31.3) |  |
|  | *ypT3* | 5 (83.3) | 1 (16.7) |  |
|  | *ypT4* | 4 (57.1) | 3 (42.9) |  |
|  |  |  |  |  |
|  | |  |  |  |
| ***Lymph node status (ypN)*** | *ypN0* | 54 (55.1) | 44 (44.9) | 0.179 |
|  | *ypN1* | 24 (68.6) | 11 (31.4) |  |
|  | *ypN2* | 11 (78.6) | 3 (21.4) |  |
|  | *ypN3* | 3 (42.9) | 4 (57.1) |  |
|  |  |  |  |  |
|  | |  |  |  |
| ***Lymph node ratio post-NACT*** | *≤0.20* | 67 (56.8) | 51 (43.2) | 0.362 |
|  | *0.21-0.65* | 20 (71.4) | 8 (28.6) |  |
|  | *>0.65* | 4 (57.1) | 3 (42.9) |  |
|  | *missing 1* |  |  |  |
|  | |  |  |  |
| ***Prognostic stage post-NACT)*** | *0* | 25 (49.0) | 26 (51.0) | **0.029** |
|  | *IA* | 38 (73.1) | 14 (26.9) |  |
|  | *IB* | 7 (100.0) | 0 (0.0) |  |
|  | *IIA* | 6 (50.0) | 6 (50.0) |  |
|  | *IIB* | 3 (42.9) | 4 (57.1) |  |
|  | *IIIA* | 7 (63.6) | 4 (36.4) |  |
|  | *IIIB* | 6 (42.9) | 8 (57.1) |  |
|  |  |  |  |  |
|  | |  |  |  |
| ***Metastasis*** | *YES* | 20 (62.5) | 12 (37.5) | 0.758 |
|  | *NO* | 72 (59.5) | 49 (40.5) |  |
|  | *missing 1* |  |  |  |
|  | |  |  |  |
| ***Proliferation index (Ki-67) pre-NACT*** | *<=20 %* | 7 (77.8) | 2 (22.2) | 0.255 |
|  | *>20 %* | 85 (58.6) | 60 (41.4) |  |
|  |  |  |  |  |
| ***Breast Tumoral response*** | *pCR* | 32 (50.8) | 31 (49.2) | 0.060 |
|  | *pPR* | 60 (65.9) | 31 (34.1) |  |
|  |  |  |  |  |
|  |  |  |  |  |
| ***Lymph nodes Response*** | *pCR* | 54 (55.1) | 44 (44.9) | 0.134 |
|  | *pPR* | 33 (71.7) | 13 (28.3) |  |
|  | *pNR* | 5 (50) | 5 (50) |  |
|  |  |  |  |  |
|  | |  |  |  |
| ***PR expression pre-NACT*** | *< 1%* | 31 (33.7) | 61 (66.3) | <**0.001** |
|  | *≥1%* | 61 (98.4) | 1 (1.6) |  |
|  | |  |  |  |
| ***AR expression pre-NACT*** | *<10%* | 4 (30.8) | 9 (69.2) | **0.013** |
|  | *≥10%* | 88 (62.9) | 52 (37.1) |  |
|  | |  |  |  |
| ***Mortality*** | *death* | 9 (47.9) | 10 (52.6) | 0.240 |
|  | *alive* | 83 (61.5) | 52 (38.5) |  |
|  |  |  |  |  |
| ***RCT post-NACT*** | *>5%* | 48 (67.6) | 23 (32.4) | 0.068 |
|  | *≤5%* | 43 (53.1) | 38 (46.9) |  |
|  | *missing 2* |  |  |  |
|  |  |  |  |  |
| ***HER2 Score pre-NACT*** | *2+* | 28 (82.4) | 6 (17.6) | **0.002** |
|  | *3+* | 64 (53.3) | 56 (46.7) |  |
|  |  |  |  |  |
| ***Proliferation index (Ki-67) post-NACT*** | *≤20 %* | 64 (64.0) | 36 (36.0) | 0.142 |
|  | *>20 %* | 28 (51.9) | 26 (48.1) |  |
|  |  |  |  |  |
|  |  |  |  |  |
| ***TIls pre-NACT*** | *<10%* | 20 (87.0) | 3 (13.0) | **0.002** |
|  | *≥10%* | 37 (51.4) | 35 (48.6) |  |
|  | *missing 59* |  |  |  |
