# Supplementary material for: Biomarker dynamics affecting neoadjuvant therapy response and outcome of HER2-positive breast cancer subtype
Source: Sci Rep. 2023 Aug 8;13:12869. doi: 10.1038/s41598-023-40071-2 (PMC10409859; doi:10.1038/s41598-023-40071-2)
Supplement: Supplementary file 4 — Supplementary Table S2. [file 41598_2023_40071_MOESM4_ESM.docx]

**Supplementary Table S2. Evaluation of hormonal receptor modifications and ki67 expression after neoadjuvant therapy in HER2 breast cancer subtype.**

The test included only pPR tumors and ER ≥1, PR ≥1 and AR ≥10. Wilcoxon rank test was used, and p value was significant at 0.05.

| Test inclusion | Variable | Mean | Median | P value |
| --- | --- | --- | --- | --- |
| All pPR | Pre-NACT KI67 | 41.0 | 38 | 0 .001 |
|  | Post-NACT KI67 | 28.0 | 23 |  |
| Only pPR and pre-NACT ER+ | Pre-NACT ER | 76.2 | 90 | 0.343 |
|  | Post-NACT ER | 78.6 | 90 |  |
| Only pPR and pre-NACT PR+ | Pre-NACT PR | 40.4 | 30 | 0.002 |
|  | Post-NACT PR | 26.9 | 15 |  |
| Only pPR and pre-NACT AR+ | Pre-NACT AR | 72.0 | 85 | 0.050 |
|  | Post-NACT AR | 67.3 | 85 |  |
