# Supplementary material for: Biomarker dynamics affecting neoadjuvant therapy response and outcome of HER2-positive breast cancer subtype
Source: Sci Rep. 2023 Aug 8;13:12869. doi: 10.1038/s41598-023-40071-2 (PMC10409859; doi:10.1038/s41598-023-40071-2)
Supplement: Supplementary file 5 — Supplementary Table S3. [file 41598_2023_40071_MOESM5_ESM.docx]

**Supplementary Table S3. Clinico-pathological data of 154 patients with HER2-positive breast cancer based on tumor localization.**

|  | | **RIGHT (n=76) *n (%)*** | **LEFT**  **(n= 75) *n (%)*** | **P value** |
| --- | --- | --- | --- | --- |
| ***Age (year)*** | *<54* | 35 (47.3) | 39 (52.7) | 0.339 |
|  | *>54* | 44 (55.0) | 36 (45.0) |  |
|  | |  |  |  |
| ***Histologic type pre-NACT,*** | *NST* | 67 (51.5) | 63 (48.5) | 0.338 |
|  | *ILC* | 3 (33.3) | 6 (66.7) |  |
|  | *APOCRINE* | 4 (44.4) | 5 (55.6) |  |
|  | *MICROPAPILLARY* | 2 (100.0) | 0 (0.0) |  |
|  | *MUCINOUS* | 0 (0.0) | 0 (0.0) |  |
|  |  |  |  |  |
|  | |  |  |  |
| ***Histologic grade pre-NACT,*** | *G2* | 10 (38.5) | 16 (61.5) | 0.151 |
|  | *G3* | 69 (53.9) | 59 (46.1) |  |
|  | |  |  |  |
| ***Tumor size (ypT)*** | *ypT0* | 40 (63.5) | 23 (36.5) | **0.001** |
|  | *ypT1a* | 11 (84.6) | 2 (15.4) |  |
|  | *ypT1b* | 7 (53.8) | 6 (46.2) |  |
|  | *ypT1c* | 7 (35.0) | 13 (65.0) |  |
|  | *ypT2* | 9 (28.1) | 23 (71.9) |  |
|  | *ypT3* | 1 (16.7) | 5 (83.3) |  |
|  | *ypT4* | 4 (57.1) | 3 (42.9) |  |
|  |  |  |  |  |
|  | |  |  |  |
| ***Lymph node status (ypN),*** | *ypN0* | 54 (55.1) | 44 (44.9) | 0.169 |
|  | *ypN1* | 19 (54.3) | 16 (45.7) |  |
|  | *ypN2* | 4 (28.6) | 10 (71.4) |  |
|  | *ypN3* | 2 (28.6) | 5 (71.4) |  |
|  |  |  |  |  |
|  | |  |  |  |
| ***Lymph node ratio post-NACT*** | *≤0.20* | 65 (55.1) | 53 (44.9) | 0.092 |
|  | *0.21-0.65* | 13 (46.4) | 15 (53.6) |  |
|  | *>0.65* | 1 (14.3) | 6 (85.7) |  |
|  | *missing 1* |  |  |  |
|  | |  |  |  |
| ***Prognostic stage post-NACT*** | *0* | 30 (58.8) | 21 (41.2) | **0.042** |
|  | *IA* | 32 (61.5) | 20 (38.5) |  |
|  | *IB* | 1 (14.3) | 6 (85.7) |  |
|  | *IIA* | 5 (41.7) | 7 (58.3) |  |
|  | *IIB* | 3 (42.9) | 4 (57.1) |  |
|  | *IIIA* | 2 (18.2) | 9 (81.8) |  |
|  | *IIIB* | 6 (42.9) | 8 (57.1) |  |
|  |  |  |  |  |
|  | |  |  |  |
| ***Metastasis*** | *YES* | 10 (31.3) | 22 (68.8) | **0.001** |
|  | *NO* | 68 (56.2) | 53 (43.8) |  |
|  | *missing 1* |  |  |  |
|  | |  |  |  |
| ***Proliferation index (Ki-67) pre-NACT*** | *≤20 %* | 6 (66.7) | 3 (33.3) | 0.342 |
|  | *>20 %* | 73 (50.3) | 72 (49.7) |  |
|  |  |  |  |  |
| ***Tumoral response*** | *pCR* | 40 (63.5) | 23 (36.5) | **0.012** |
|  | *pPR* | 39 (42.9) | 52 (57.1) |  |
|  |  |  |  |  |
|  |  |  |  |  |
| ***Lymph nodes Response*** | *pCR* | 54 (55.1) | 44 (44.9) | 0.104 |
|  | *pPR* | 23 (50.0) | 23 (50.0) |  |
|  | *pNR* | 2 (20.0) | 8 (80.0) |  |
|  |  |  |  |  |
| ***ER expression pre-NACT,*** | *<1%* | 31 (50.0) | 31 (50.0) | 0.791 |
|  | *≥1%* | 48 (52.2) | 44 (47.8) |  |
|  | |  |  |  |
| ***PR expression pre-NACT,*** | *< 1%* | 45 (48.9) | 47 (51.1) | 0.471 |
|  | *≥1%* | 34 (54.8) | 28 (45.2) |  |
|  | |  |  |  |
| ***AR expression pre-NACT*** | *<10%* | 7 (50.0) | 7 (50.0) | 0.919 |
|  | *≥10%* | 72 (51.4) | 68 (48.6) |  |
|  | |  |  |  |
| ***Mortality*** | *death* | 5 (26.3) | 14 (73.7) | **0.020** |
|  | *alive* | 74 (54.8) | 61 (45.2) |  |
|  |  |  |  |  |
| ***RCT post-NACT*** | *>5%* | 23 (32.4) | 48 (67.6) | **0.001** |
|  | *≤5%* | 56 (69.1) | 25 (30.9) |  |
|  | *missing 2* |  |  |  |
|  |  |  |  |  |
| ***HER2 Score pre-NACT*** | *2+* | 16 (47.1) | 18 (52.9) | 0.575 |
|  | *3+* | 63 (52.5) | 57 (47.5) |  |
|  |  |  |  |  |
| ***TILS pre-NACT*** | *<10%* | 12 (52.2) | 11 (47.8) | 0.948 |
|  | *≥10%* | 37 (51.4) | 35 (48.6) |  |
|  | *missing 59* |  |  |  |
|  |  |  |  |  |
| ***Proliferation index (Ki-67) post-NACT*** | *≤20 %* | 53 (53.0) | 47 (47.0) | 0.565 |
|  | *>20 %* | 26 (48.1) | 28 (51.9) |  |
|  |  |  |  |  |
