# Supplementary material for: Biomarker dynamics affecting neoadjuvant therapy response and outcome of HER2-positive breast cancer subtype
Source: Sci Rep. 2023 Aug 8;13:12869. doi: 10.1038/s41598-023-40071-2 (PMC10409859; doi:10.1038/s41598-023-40071-2)
Supplement: Supplementary file 6 — Supplementary Table S4. [file 41598_2023_40071_MOESM6_ESM.docx]

**Supplementary Table S4. Performance metrics for the considered classification methods.**

We tested the following algorithms: k-Nearest Neighbor (k-NN), Simple Logistic (SL), Support Vector Machine (SVM), Multi-Layer Perceptron (MLP), Random Forest (RF).

| **Classifier** | **Accuracy** | **Sensitivity** | **Specificity** | **AUC** |
| --- | --- | --- | --- | --- |
| MLP | 69.8% | 0.705 | 0.691 | 0.726 |
| RF | 69.8% | 0.672 | 0.721 | 0.768 |
| RF (1000 trees) | 71.3% | 0.656 | 0.765 | 0.779 |
| SL | 71.3% | 0.738 | 0.691 | 0.788 |
| SVM-linear (SMO) | 71.3% | 0.721 | 0.706 | 0.714 |
| SVM-linear (SGD) | 74.4% | 0.754 | 0.735 | 0.745 |
| k-NN (k = 9) | 76.0% | 0.836 | 0.691 | 0.818 |
| k-NN (k = 7) | 78.3% | 0.803 | 0.765 | 0.814 |
| k-NN (k = 5) | 79.1% | 0.770 | 0.809 | 0.822 |
