# Supplementary material for: Biomarker dynamics affecting neoadjuvant therapy response and outcome of HER2-positive breast cancer subtype
Source: Sci Rep. 2023 Aug 8;13:12869. doi: 10.1038/s41598-023-40071-2 (PMC10409859; doi:10.1038/s41598-023-40071-2)
Supplement: Supplementary file 7 — Supplementary Table S5. [file 41598_2023_40071_MOESM7_ESM.docx]

Supplementary Table S5. Correlation between clinico-pathological parameters in HER2-positive breast cancer treated with neoadjuvant therapy

Spearman’s rho was used. * p < .05, ** p < .01, *** p < .001
